# Enhanced CT Textures Derived From Computer Mathematic Distribution Analysis Enables Arterial Enhancement Fraction Being an Imaging Biomarker Option of Hepatocellular Carcinoma

**DOI:** 10.3389/fonc.2020.01337

**Published:** 2020-08-11

**Authors:** Xiaonan Mao, Yan Guo, Zaiming Lu, Feng Wen, Hongyuan Liang, Wei Sun

**Affiliations:** ^1^Department of Radiology, ShengJing Hospital of China Medical University, Shenyang, China; ^2^GE Healthcare, Shanghai, China

**Keywords:** texture, AEF, AFP, heterogeneity, angiogenesis, HCC

## Abstract

**Purpose:** This study aims to explore the imaging–clinic relationship and an optional imaging biomarker of hepatocellular carcinoma (HCC) by using texture analysis on arterial enhancement fraction (AEF).

**Materials and Methods:** The HCC patients treated in No. 2 Interventional Ward, ShengJing Hospital of China Medical University from June 2018 to June 2019 were enrolled, for whom tri-phasic enhanced CT scans were acquired. Perfusion analysis and texture analysis were then performed on the tri-phasic enhanced CT images. After the region of interest (ROI) of viable HCC was drawn, 13 AEF textures describing the values distribution were conducted. A between-groups comparison of AEF textures was made where the cases had grouping properties, a correlation analysis was made between AEF textures and alpha-fetoprotein (AFP) as well as other clinical data which were digital, and regression analysis was made when a significant correlation was found. SPSS 19.0 (IBM) was utilized for statistical analysis; a significant difference was considered when *P* < 0.05.

**Results:** Twenty-five HCC patients were enrolled. Several AEF textures were found to have a correlation with clinical features, including previous surgery history, age, glutamic oxaloacetylase, indirect bilirubin, creatinine, and AFP. The majority of AEF textures (up to 9/13) were found to have a correlation with AFP (SD, variance, uniformity, energy, entropy, inertia, correlation, inverse difference moment, and cluster prominence), while six or seven textures have a linear or cubic relationship with AFP (SD, variance, uniformity, inertia, correlation, cluster prominence, plus inverse difference moment).

**Conclusion:** The AEF textures of HCC are strongly correlated with and are impacted by AFP, which may enable AEF to act as an optional imaging biomarker of HCC.

## Introduction

Hepatocellular carcinoma (HCC) is one of the most common cancers worldwide, accounting for 90% of primary malignant liver neoplasms (1). In China, the situation is almost the same; 85–90% of primary liver cancers are HCCs (2). An early and precise detection is vital in the diagnosis and follow-up of HCC, where the imaging finding featuring a unique enhancement pattern is acknowledged as a great help, no matter if in China (2), Europe (3), or America (4).

The liver is fed by both the portal vein (75%) and the hepatic artery (25%). As HCC develops, portal feeding decreases, while arterial feeding increases and becomes more and more predominant (5–8). The changing of perfusion proportion is a unique histological feature of HCC, which can be reflected by arterial enhancement fraction (AEF) (9), the ratio between hepatic artery perfusion and portal vein perfusion (10–14). AEF can be obtained based on routine tri-phasic enhanced CT images by using the formula CTa–Ctu/CTp–CTu (where CTa is the CT value in arterial phase, CTp is the CT value in portal phase, and CTu is the unenhanced CT value), which means that extra contrast or radiation exposure can be avoided. At the same time, AEF can define the viable tumor by depicting the region with perfusion, which means that non-tumorous tissue like calcification, necrosis, or lipiodol accumulation can be avoided.

Considering that the changing of blood feeding is heterogeneous inside the tumor, an overall AEF covering the whole tumor may not describe the inherent details of HCC. Texture analysis had been widely applied in medical imaging as reported (15–19), which enables a mathematical and statistical description of an image by evaluating the distribution of pixels. The present study performed texture analysis on AEF, along with clinical data extraction, with the aim to explore the imaging–clinic relationship and an optional imaging biomarker of hepatocellular carcinoma. A good coordination between clinicians and imaging engineers is necessary to guarantee the practicability of this study.

## Materials and Methods

### Patient Enrollment

**Figure 1:**
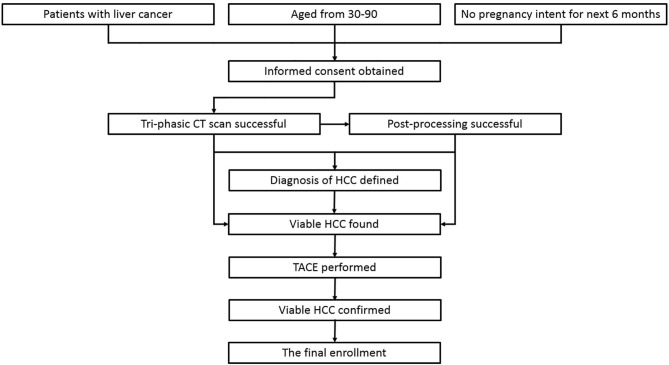
The route of case enrollment. The cases which failed in any step of the route would be excluded from the study.

The cases enrolled in this study were HCC patients who were treated in No. 2 Interventional Ward, ShengJing Hospital of China Medical University from June 2018 to June 2019. Approved by the institutional ethical committee of our center, the enrollment was achieved *via* the following route (Figure 1): (1) patients with liver cancer, (2) age from 30–90 years old, (3) not intending to be pregnant in the next 6 months, (4) written informed consent was obtained, (5) tri-phasic enhanced CT scan was performed, (6) the quality of the images was satisfactory for post-processing, (7) the diagnosis of HCC was defined clinically or pathologically, (8) viable HCC was found based on CT and/or post-processing results, (9) transarterial chemoembolization (TACE) was subsequently performed to treat the patients, and (10) the viable HCC was confirmed by hepatic arteriography during TACE. Cases which failed in any step of the route would be excluded. Besides that, cases should be excluded when any of the following situations happened: (1) images with motion artifacts causing a difficulty in the region of interest (ROI) drawing, (2) abnormal density artifacts involving ROI, (3) unmatched slices between phases even though 3D non-rigid motion registration was applied, (4) diffuse HCC, (5) tiny HCC (<1 cm), (6) big fistula between vessels found during TACE, (7) any later evidence against the diagnosis, and (8) patients asking for quit.

Age, weight, gender, hepatitis type, alcoholic background, and family history were recorded. The imaging features related to liver cirrhosis were reviewed by two radiologists with at least 5 years of work experience, including cirrhotic deformation, ascites, varices, splenomegaly, and hepatic encephalopathy. All cases were then divided into three degrees (absent, mild, and severe) based on the imaging findings. Additionally, some lab indexes involving liver function, renal function, coagulation function, ammonia, and alpha-fetoprotein (AFP) were gathered. Barcelona Staging System (3), China Staging System (2), and Child–Pugh Scoring System were used for the final classification of the enrolled cases.

### Image Processing

CT scan was acquired with a 128-row multi-detector CT (iCT 256, Philips, Netherlands). The scanning parameters were as follows: tube voltage, 100 kVp, with automatic tube current modulation; pitch, 0.993; rotation time, 0.5 s; collimation 128 × 0.635; field of view, 350 × 350 mm; and slice thickness, 3 mm. Tri-phasic enhanced images were acquired after the bolus injection of iodixanol (Visipague 270, GE, Ireland). The volume of contrast used was calculated as 1.2 ml/kg body weight, and the injection rate was 4.5 ml/s, followed by 20 ml of saline flush. The acquisition times for each phase were arterial phase 23 s, portal phase 45 s, and delay phase 120 s, which were determined by pre-experiments where the acquisition time matched the three phases in the majority of the patients.

The unenhanced and tri-phasic enhanced CT images (DCM format) were loaded into C.K. Software (CT-Kinetics, GE Healthcare, China) for the analysis based on a liver model. 3D non-rigid motion registration was applied for each data set before analysis to overcome the complicated movement of the liver during breathing. The aorta was chosen as the input artery and the portal vein as the input vein, and the time–density curve was obtained. The parametric perfusion maps of AEF were generated automatically. A lesion ROI was delineated around the tumor outline for the largest cross-sectional area based on both AEF map and CT map that can best show the outline of the tumor. All the necrosis, calcification, and lipiodol accumulation should be excluded. It is acceptable to shrink the tumor region a little smaller than it is shown in order to guarantee that the whole ROI was tumorous. Two radiologists did the ROI drawing work with an agreement to make sure that the final ROI was correct. Another round of ROI on the liver parenchyma was also placed as control. Then, the AEF of each pixel within the lesion ROI was calculated based on the AEF map. The entire texture analysis was performed using the C.K. software automatically. A total of 13 textures showing the mathematic distribution of AEF were generated, including mean value, SD, variance, skewness, kurtosis, uniformity, energy, entropy, inertia, correlation, inverse difference moment, cluster shade, and cluster prominence.

### Data Statistics

Each AEF texture group (13 groups) should be tested with Kolmogorov–Smirnov test to judge whether they were accorded with normal distribution prior to the statistical analysis. Then, first, a between-groups comparison of AEF textures was made, wherein the cases had grouping properties such as gender, hepatitis type, cirrhotic degree, Barcelona stage, Child–Pugh score, etc. Second, a correlation analysis was made between AEF textures and some clinical data which were digital, such as age, weight, albumin, bilirubin, AFP, etc. Third, for the reason that the AFP and the AEF textures were both some sort of reflection of the HCCs' inherent attributes, a regression analysis was made when a significant correlation was found to determine the causal relationship between them. SPSS 19.0 (IBM) was utilized for statistical analysis. A significant difference was considered when *P* < 0.05.

## Results

### Patient Enrollment

Sixty-nine patients were initially involved in this study. Informed consent was obtained from all of them. However, eight patients did not pass the CT scan because of either equipment malfunction or personal condition. Two cases were diagnosed to be intrahepatic cholangiocarcinoma and liver metastases and were judged as failed in the enrollment. The other 59 patients were all clinically diagnosed with HCC; some of them were defined by biopsy. After a systemic assessment, therapeutic recommendations were given. A total of 28 patients accepted TACE for tumor control; the others were tumor-free or unable/unwilling to have TACE due to multiple reasons like physical condition, fare, or risk. TACE was performed successfully in 28 patients, with no complications observed. The viable HCCs were confirmed during hepatic arteriography. Two patients were excluded for the reason of diffuse HCC and big arterioportal fistula. Additionally, one patient asked to quit the study for personal reasons. Therefore, 25 cases were finally enrolled, for whom the baseline characteristics are listed in Table 1.

**Table 1:** Baseline of the enrolled cases.

**Item**	**Mean ± SD coverage or number**
Sample size	25
Age	61.80 ± 9.53, 46–84 years
Weight	68.40 ± 12.40, 50–90 kg
Gender	Male (*n* = 21); female (*n* = 4)
Hepatitis type	B (*n* = 20); C (*n* = 3); no hepatitis (*n* = 2)
Alcoholic background	Yes (*n* = 12); no (*n* = 13)
HCC family history	Yes (*n* = 5); no (*n* = 20)
Previous surgery	Yes (*n* = 6); no (*n* = 19)
Previous TACE	Yes (*n* = 24); no (*n* = 1)
Diagnosis defined	Pathologically (*n* = 6); clinically (*n* = 19^a^)
Liver cirrhotic deformation	Absent (*n* = 5); mild (*n* = 13); severe (*n* = 7)
Ascites	Absent (*n* = 15); mild (*n* = 9); severe (*n* = 1)
Varices	Absent (*n* = 10); mild (*n* = 8); severe (*n* = 7)
Splenomegaly^b^	Absent (*n* = 7); mild (*n* = 11); severe (*n* = 6)
Hepatic encephalopathy	Absent (*n* = 25); mild (*n* = 0); severe (*n* = 0)
Barcelona stage	A (*n* = 11); B (*n* = 7); C (*n* = 7); D (*n* = 0)
China stage	Ia (*n* = 5); Ib (*n* = 6); IIa (*n* = 3); IIb (*n* = 4); IIIa (*n* = 2); IIIb (*n* = 5); IV (*n* = 0)
Child–pugh score	A5 (*n* = 11); A6 (*n* = 4); B7 (*n* = 6); B8 (*n* = 3); B9 (*n* = 1); C10 (*n* = 0)
Albumin	36.00 ± 6.42, 21.1–48.2 g/L
Ammonia	65.74 ± 19.64, 37.0–106.7 mmol/L
Alanine aminotransferase	35.52 ± 16.35, 12–85 g/L
Glutamic oxaloacetylase	48.92 ± 25.18, 24–122 g/L
Direct bilirubin	9.016 ± 5.70, 2.3–30.6 mmol/L
Indirect bilirubin	11.28 ± 5.28, 3.8–24.3 mmol/L
Urea nitrogen	5.53 ± 1.46, 3.10–8.43 U/L
Creatinine	70.67 ± 14.61, 39.9–99.3 U/L
Prothrombin time	13.02 ± 1.60, 10.9–15.9 S
Fibrinogen	2.35 ± 0.70, 1.01–3.71 g/L
AFP	863.4748 ± 2,308.13, 2.1–11,041 μg/L (*n* = 25); 264.3422 ± 371.88, 2.1–1,186 μg/L (*n* = 23^c^)

a One case in this group was judged to be false-negative despite that the biopsy found no malignant cells because of the possibility of an unsuccessful access to the real tumorous tissue during the procedure, which was clinically diagnosed to be HCC according to the current guideline.

b One patient had a history of splenectomy.

c *AFP could be measured in all 25 cases, but the values in two cases were so much higher than the others and caused the values to be extremely dispersed. This situation should be considered in correlation and regression analysis*.

### Image Processing

**Figure 2:**
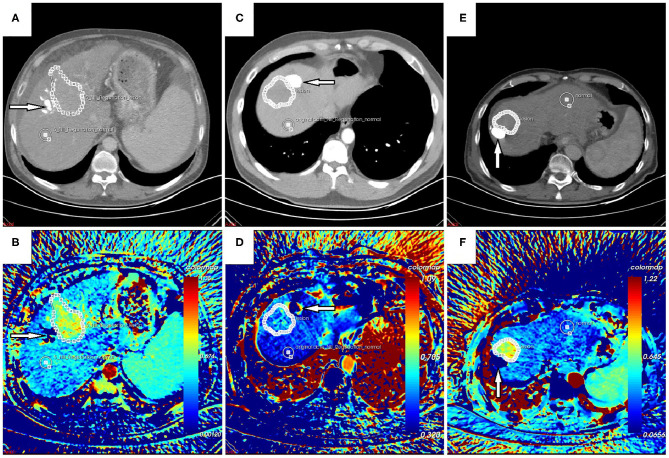
**(A,B)** Follow-up CT scan and arterial enhancement fraction (AEF) map of a 54-year-old male patient with no hepatitis background. **(C,D)** Follow-up CT scan and AEF map of a 48-year-old male patient with hepatitis B. **(E,F)** Follow-up CT scan and AEF map of a 66-year-old male patient with hepatitis B. They were all treated with transarterial chemoembolization (TACE) previously. The previous lesion with lipiodol deposition could be seen (arrow) on CT images, which should show as a “hollow zone” **(B,F)** on the AEF map despite some fake color **(D)** that was occasionally present due to tiny motion. Beside the “hollow zone,” a warmer region could be detected and was delineated as the “lesion” region of interest (ROI) even though it was difficult to recognize on CT images. Another ROI of “control” was delineated as well. The AEF mean value was significantly higher for “lesion” than for “control,” which indicated tumor recurrence, and they were confirmed by hepatic arteriography during the following TACE.

**Figure 3:**
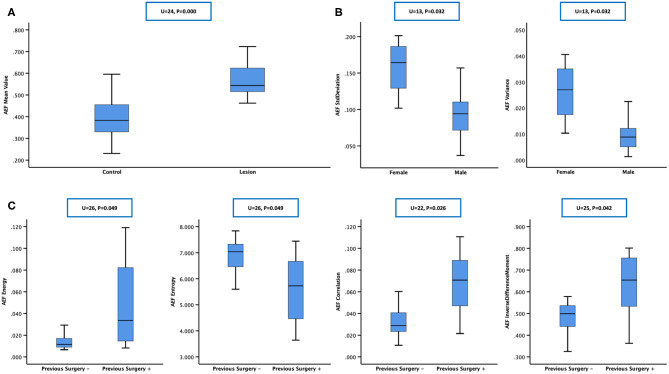
Mann–Whitney test was chosen for between-group statistical analysis. **(A)** The arterial enhancement fraction (AEF) mean value of “lesion” was higher than that of “control,” which meant that hepatocellular carcinomas (HCCs) had a higher AEF than normal liver parenchyma, indicating an obvious tumorous angiogenesis present in HCC, with arterial/portal feeding proportion rising up. **(B)** Box plot showing that SD and variance of AEF were higher in females, showing that the heterogeneity of HCC's feeding proportion might be bigger in women, which indicated that the process of tumor angiogenesis might be more complicated in women in this study. **(C)** Box plot showing that energy, correlation, and inverse difference moment of AEF were higher while entropy was lower in patients having a previous surgery history, showing that the heterogeneity of HCC's feeding proportion might be smaller in previous surgery-treated patients, which indicated that the process of tumor angiogenesis in the recurrent HCCs after curative surgery might be less complicated than the other primary ones in this study.

Each CT scan took about 5 min. No accident occurred, no discomfort was reported, and no contrast-related complications arose. The image quality was satisfactory, and post-processing was accomplished. In general, the color of the “lesion” was warmer than that of the “control” on the AEF map (Figure 2). For patients who had been treated with TACE previously, the embolized region with lipiodol accumulation had no blood perfusion. This region should show as a “hollow zone” on the AEF map. When any abnormal color was recognized beside the “hollow zone.” Tumor recurrence should be considered. After the viable tumor region was delineated by hand, 13 textures were all extracted for all 25 cases. The Kolmogorov–Smirnov test showed that some textures fitted normal distribution while some did not. To ensure the creditability of the following statistical analysis, non-parametric tests were used. The results showed that the AEF mean value was, respectively, 0.578 ± 0.102 (range, 0.463–0.913) for “lesion” and 0.389 ± 0.082 (range, 0.223–0.595) for “control.” The difference was significant based on Mann–Whitney test (*U* = 24; *P* = 0.000 < 0.05; Figure 3A).

### Data Statistics

#### Group Comparison

According to the baseline of enrolled cases, the patients could be grouped based on gender (male vs. female), hepatitis type (B vs. C vs. others), alcoholic background (yes vs. no), HCC family history (yes vs. no), previous surgery history (yes vs. no), liver cirrhotic deformation level (absent vs. mild vs. severe), ascites level (absent vs. mild vs. severe), varices level (absent vs. mild vs. severe), splenomegaly level (absent vs. mild vs. severe), Barcelona stage (A vs. B vs. C), China stage (I vs. II vs. III), and Child–Pugh level (A vs. B). Mann–Whitney test was chosen for two groups, and Kruskal–Wallis test was chosen for three or more groups. The results are listed in Table 2, from which we could see that (1) SD and variance of AEF were lower in males and higher in females (Figure 3B) and (2) energy, correlation, and inverse difference moment of AEF were higher, while entropy was lower in patients having a previous surgery history (Figure 3C).

**Table 2:** Results of the group comparison.

**Grouping item**	**The AEF textures with statistical significance**	**Mean ± SD**
Gender (male vs. female)	SD (*U* = 13; *P* = 0.032)	0.098 ± 0.039 vs. 0.159 ± 0.042
	Variance (*U* = 13; *P* = 0.032)	0.011 ± 0.009 vs. 0.026 ± 0.013
Previous surgery history^a^ (yes vs. no)	Energy (*U* = 26; *P* = 0.049)	0.049 ± 0.044 vs. 0.014 ± 0.007
	Entropy (*U* = 26; *P* = 0.049)	5.612 ± 1.435 vs. 6.898 ± 0.623
	Correlation (*U* = 22; *P* = 0.026)	0.068 ± 0.032 vs. 0.035 ± 0.022
	Inverse difference moment (*U* = 25; *P* = 0.042)	0.627 ± 0.168 vs. 0.480 ± 0.076
Hepatitis type (B vs. C vs. others)	None	
Alcoholic background (yes vs. no)	None	
HCC family history (yes vs. no)	None	
Liver cirrhotic deformation level (absent vs. mild vs. severe)	None	
Ascites level (absent vs. mild vs. severe)	None	
Varices level (absent vs. mild vs. severe)	None	
Splenomegaly level (absent vs. mild *vs* severe)	None	
Barcelona stage (A vs. B vs. C)	None	
China stage (I vs. II vs. III)	None	
Child–pugh level (A vs. B)	None	

a *The patient had undergone curative surgery to treat HCC before this enrollment. Only the textures with significant differences between groups were listed in this table. The results showed that the heterogeneity of HCC's feeding proportion might be bigger in women and smaller in previously surgery-treated patients*.

#### Correlation Analysis

**Figure 4:**
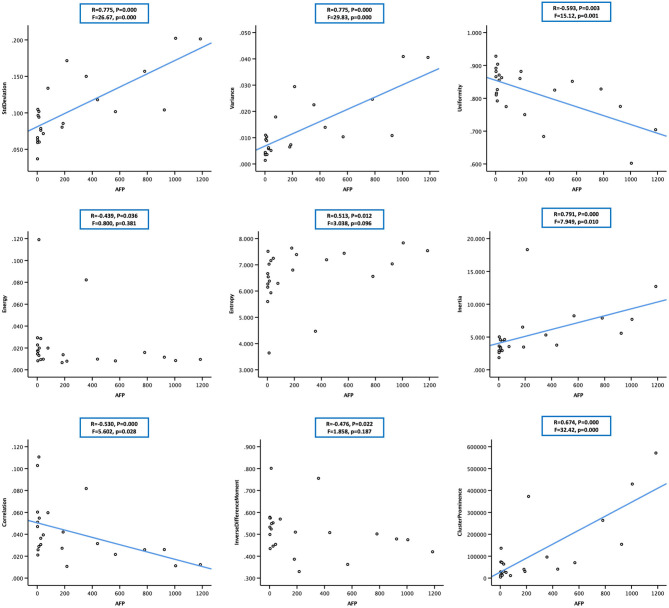
Scatter plot showing the correlation between alpha-fetoprotein (AFP; in 23 cases) and nine arterial enhancement fraction (AEF) textures. Except for energy, entropy, and inverse different moment, a linear relationship could be obtained between AFP and the other six textures. The blue line indicated a positive two-way linear causal relationship between AFP (if not too high) and AEF heterogeneity, which meant that a moderate AFP secretion and the complexity of HCC's angiogenesis might have impact on each other in HCC.

According to the baseline of enrolled cases, the digital clinical data included age, weight, albumin, ammonia, alanine aminotransferase, glutamic oxaloacetylase, direct bilirubin, indirect bilirubin, urea nitrogen, creatinine, prothrombin time, fibrinogen, and AFP. Spearman correlation analysis was used for statistical analysis. The results are listed in Table 3, from which we could see that (1) the AEF textures having a correlation with age (6/13) were the same as creatinine (6/13), but reverse; (2) some AEF textures had a correlation with glutamic oxaloacetylase (3/13) rather than with alanine aminotransferase (0/13); (3) more AEF textures had a correlation with indirect bilirubin (6/13) than with direct bilirubin (1/13); and (4) most AEF textures (up to 9/13) had a correlation with AFP (Figure 4).

**Table 3:** Results of the correlation analysis.

**Clinical items**	**Correlated textures (25 cases)**	**Correlated textures (23 cases)**
Age	SD (*R* = 0.484; *P* = 0.014)	
	Variance (*R* = 0.484; *P* = 0.014)	
	Uniformity (*R* = −0.430; *P* = 0.032)	
	Inertia (*R* = 0.493; *P* = 0.012)	
	Correlation (*R* = −0.483; *P* = 0.014)	
	Cluster prominence (*R* = 0.651; *P* = 0.000)	
Glutamic oxaloacetylase	Energy (*R* = −0.459; *P* = 0.021)	
	Entropy (*R* = 0.474; *P* = 0.017)	
	Correlation (*R* = −0.466; *P* = 0.019)	
Direct bilirubin	Skewness (*R* = −0.418; *P* = 0.038)	
Indirect bilirubin	Skewness (*R* = −0.425; *P* = 0.034)	
	Energy (*R* = −0.425; *P* = 0.034)	
	Entropy (*R* = 0.415; *P* = 0.039)	
	Correlation (*R* = −0.467; *P* = 0.019)	
	Inverse different moment (*R* = −0.406; *P* = 0.044)	
	Cluster shade (*R* = −0.407; *P* = 0.044)	
Creatinine	SD (*R* = −0.563; *P* = 0.003)	
	Variance (*R* = −0.563; *P* = 0.003)	
	Uniformity (*R* = 0.624; *P* = 0.001)	
	Inertia (*R* = −0.412; *P* = 0.041)	
	Correlation (*R* = 0.612; *P* = 0.001)	
	Cluster prominence (*R* = −0.776; *P* = 0.000)	
AFP	SD (*R* = 0.762; *P* = 0.000)	^*^SD (*R* = 0.755; *P* = 0.000)
	Variance (*R* = 0.762; *P* = 0.000)	^*^Variance (*R* = 0.755; *P* = 0.000)
	Uniformity (*R* = −0.658; *P* = 0.000)	^*^Uniformity (*R* = −0.593; *P* = 0.003)
	Inertia (*R* = 0.692; *P* = 0.000)	^*^Energy (*R* = −0.439; *P* = 0.036)
	Correlation (*R* = −0.441; *P* = 0.027)	^*^Entropy (*R* = 0.513; *P* = 0.012)
	Inverse difference moment (*R* = −0.398; *P* = 0.049)	^*^Inertia (*R* = 0.791; *P* = 0.000)
	Cluster prominence (*R* = 0.632; *P* = 0.001)	^*^Correlation (*R* = −0.530; *P* = 0.009)
		^*^Inverse difference moment (*R* = −0.476; *P* = 0.022)
		^*^Cluster prominence (*R* = 0.674; *P* = 0.000)
Weight, albumin, ammonia, prothrombin time, alanine aminotransferase, urea nitrogen, fibrinogen	None	

AFP could be measured in all 25 cases, but the values in two cases were so much higher than the others and caused the values to be extremely dispersed. Correlation analysis was performed twice, respectively, with these two cases included and excluded (marked with “*”). Only the textures having significant correlations with clinical data were listed in this table. The R-value referred to the correlation intensity and direction (positive or negative). The results showed that the heterogeneity of HCC's feeding proportion or we could say that the complexity of HCC's angiogenesis might be moderately positively correlated with age while negatively correlated with creatinine, slightly positively correlated with glutamic oxaloacetylase, moderately positively correlated with indirect bilirubin, and strongly positively correlated with AFP.

#### Regression Analysis

**Figure 5:**
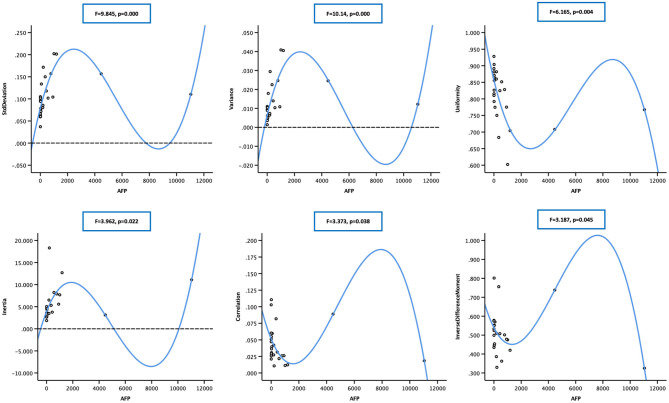
Scatter plot showing the cubic relationship between alpha-fetoprotein (AFP; in 25 cases) and correlated arterial enhancement fraction (AEF) textures (cluster prominence not included). The blue line indicated a one-way positive cubic causal relationship between AFP (if too high) and AEF heterogeneity, which meant that an intense AFP secretion might have a strong impact on the complexity of HCC's angiogenesis.

Two-way regression analysis was made between AEF textures and AFP since correlations were found in “Correlation Analysis” in order to confirm the cause-and-effect relationship between them. Given that part of AEF textures and AFP neither fitted normal distribution by Kolmogorov–Smirnov test, linear, quadratic, and cubic regression were all performed. The results are listed in Table 4, from which we could see that (1) if the two cases with much higher AFP were excluded, there was often (6/9) a two-way causal relationship between AFP and the correlated AEF textures in linear regression (Figure 4) and (2) if all cases were included, there was always (7/7) a one-way causal relationship between AFP and the correlated AEF textures in cubic regression (Figure 5).

**Table 4:** Two-way regression results between AFP and correlated AEF textures.

	**23 cases**			**25 cases**		
	**Linear**	**Quadratic**	**Cubic**	**Linear**	**Quadratic**	**Cubic**
**Regression with AFP as independent variable**						
SD	*F* = 26.67; *P* = 0.000	*F* = 12.77; *P* = 0.000	*F* = 10.70; *P* = 0.000	*F* = 1.062; *P* = 0.313	*F* = 6.035; *P* = 0.008	*F* = 9.845; *P* = 0.000
Variance	*F* = 29.83; *P* = 0.000	*F* = 14.52; *P* = 0.000	*F* = 12.47; *P* = 0.000	*F* = 0.688; *P* = 0.415	*F* = 5.828; *P* = 0.009	*F* = 10.14; *P* = 0.000
Uniformity	*F* = 15.12; *P* = 0.001	*F* = 7.253; *P* = 0.004	*F* = 5.123; *P* = 0.009	*F* = 2.446; *P* = 0.131	*F* = 5.675; *P* = 0.010	*F* = 6.165; *P* = 0.004
Energy	*F* = 0.800; *P* = 0.381	*F* = 0.400; *P* = 0.675	*F* = 0.255; *P* = 0.857			
Entropy	*F* = 3.038; *P* = 0.096	*F* = 1.454; *P* = 0.257	*F* = 0.980; *P* = 0.423			
Inertia	*F* = 7.949; *P* = 0.010	*F* = 3.951; *P* = 0.036	*F* = 4.846; *P* = 0.011	*F* = 2.498; *P* = 0.128	*F* = 1.233; *P* = 0.311	*F* = 3.962; *P* = 0.022
Correlation	*F* = 5.602; *P* = 0.028	*F* = 2.678; *P* = 0.051	*F* = 1.736; *P* = 0.052	*F* = 0.272; *P* = 0.607	*F* = 0.240; *P* = 0.789	*F* = 3.373; *P* = 0.038
Inverse difference moment	*F* = 1.858; *P* = 0.187	*F* = 0.919; *P* = 0.415	*F* = 0.732; *P* = 0.547	*F* = 1.015; *P* = 0.324	*F* = 1.494; *P* = 0.246	*F* = 3.187; *P* = 0.045
Cluster prominence	*F* = 32.42; *P* = 0.000	*F* = 21.33; *P* = 0.000	*F* = 17.03; *P* = 0.000	*F* = 0.125; *P* = 0.727	*F* = 1.773; *P* = 0.193	*F* = 8.812; *P* = 0.001
**Regression with AFP as dependent variable**						
SD	*F* = 26.67; *P* = 0.000	*F* = 14.24; *P* = 0.000	*F* = 9.681; *P* = 0.000	*F* = 1.062; *P* = 0.313	*F* = 0.964; *P* = 0.397	*F* = 0.652; *P* = 0.590
Variance	*F* = 29.83; *P* = 0.000	*F* = 14.57; *P* = 0.000	*F* = 10.83; *P* = 0.000	*F* = 0.688; *P* = 0.415	*F* = 0.982; *P* = 0.390	*F* = 0.730; *P* = 0.546
Uniformity	*F* = 15.12; *P* = 0.001	*F* = 7.330; *P* = 0.004	*F* = 7.332; *P* = 0.004	*F* = 2.446; *P* = 0.131	*F* = 1.669; *P* = 0.211	*F* = 1.628; *P* = 0.219
Energy	*F* = 0.800; *P* = 0.381	*F* = 0.869; *P* = 0.435	*F* = 1.451; *P* = 0.259			
Entropy	*F* = 3.038; *P* = 0.096	*F* = 3.030; *P* = 0.071	*F* = 3.249; *P* = 0.060			
Inertia	*F* = 7.949; *P* = 0.010	*F* = 16.51; *P* = 0.000	*F* = 15.63; *P* = 0.000	*F* = 2.498; *P* = 0.128	*F* = 2.298; *P* = 0.124	*F* = 3.556; *P* = 0.032
Correlation	*F* = 5.602; *P* = 0.028	*F* = 5.669; *P* = 0.011	*F* = 4.665; *P* = 0.013	*F* = 0.272; *P* = 0.607	*F* = 0.571; *P* = 0.573	*F* = 0.681; *P* = 0.573
Inverse difference moment	*F* = 1.858; *P* = 0.187	*F* = 1.016; *P* = 0.380	*F* = 1.059; *P* = 0.365	*F* = 1.105; *P* = 0.324	*F* = 3.785; *P* = 0.039	*F* = 3.785; *P* = 0.039
Cluster prominence	*F* = 32.42; *P* = 0.000	*F* = 15.63; *P* = 0.000	*F* = 11.31; *P* = 0.000	*F* = 0.125; *P* = 0.727	*F* = 0.394; *P* = 0.679	*F* = 0.857; *P* = 0.479

*When two cases with high AFP were excluded, the AFP values were relatively concentrated; then, the relationship between AFP and AEF textures was often significant, even using a different regression pattern. We could also notice that they had a double-way causal relationship in linear regression, indicating that they might have impact on each other. When all the cases were included, the AFP values were relatively dispersed; then, the relationship between AFP and AEF textures was barely significant overall, but always significant only when using cubic regression. We should also notice that there was only a one-way causal relationship between them when AFP was the independent variable in cubic regression, indicating that AFP might have impact on AEF heterogeneity*.

## Discussion

AEF is a perfect indicator that reflects the perfusion proportion of HCC between the hepatic artery and the portal vein. In this study, AEF was chosen to be analyzed instead of CT value because (9–14) (1) it is the valid perfusion that feeds the tumor to be viable, which means that the real tumor region should be delineated by a perfusion map; (2) the changing of perfusion proportion is one of the unique characteristics throughout HCC's generation and development; so AEF is not only a perfusion parameter but also a biomarker of HCC; and (3) post-processing technology enables the perfusion analysis based on routine tri-phasic enhanced CT images and ensures that there is no more contrast injection and radiation exposure. Texture analysis is a good method in medical imaging analysis. Several mostly used textures had been described in literatures (15–19). For better understanding in this study, uniformity, energy, inertia, correlation, and inverse difference moment quantified the homogeneity of AEF, entropy, cluster shade, and cluster prominence. SD and variance quantified the heterogeneity of AEF, and skewness and kurtosis quantified the match between AEF distribution and normal distribution as well.

Chronic hepatitis and liver cirrhosis are the basis before HCC develops, which may have many different causes as known. We made comparisons between different cirrhotic baseline characteristics with the aim to investigate their impacts on AEF. Our results did find an increase of AEF SD and variance in women; however, it was more likely to be a coincidence than a regularity considering the insufficient textures showing a difference and the big inequality of the group size. The number of women in this study was only four, which could easily make the data more dispersed compared with those of 21 men. At the same time, the previous curative surgery history group of six cases had a difference in four textures, indicating that their AEF was more homogeneous. It is perhaps because the post-surgery follow-up was so regular and frequent that a recurrent HCC could be found at a small size or an early stage, while the other 19 cases involved multiple sizes, shapes, and appearance as a result of long and complicated tumor development, which could probably be the reasons causing the AEF to be more heterogeneous. Our results also suggested that age might have a moderate positive correlation with AEF heterogeneity. That was to say, the process of HCCs' angiogenesis might be more complicated in older patients, which should be correlated with longer disease history and more risk exposure.

Creatinine is an index to evaluate renal function. Our study found a moderate negative correlation between creatinine and AEF heterogeneity. Studies on such relationship are very rare. In 2016, Shao (20) reported a finding of the correlation between serum vascular endothelial growth factor and renal function. They explained that abnormal angiogenesis could cause the formation of immature blood vessels (20). Another research (21) also proved that the increased VEGF expression could promote abnormal blood vessel formation in diabetic kidney disease. These studies indeed inspired us about the possible negative correlation between angiogenesis and renal function, which needs bigger-sized and specifically designed studies to prove. Not like creatinine, indirect bilirubin is an index to evaluate liver function. It is supposed to be converted to direct bilirubin after some biochemical reaction conducted by liver cells so it can be used to reflect the metabolic ability of liver. Our results suggested that indirect bilirubin had a moderate positive correlation with AEF heterogeneity. Such finding was also barely seen in literatures. Youssry (22) described sickle cell disease in his study. They found that indirect bilirubin was an independent predictor of sFLT-1 that had an anti-angiogenic effect (23), and there was a significant positive correlation between them (22). Their finding seemed to be contrary to ours, so we can infer that their relationship must be more complexed than that shown in our study. Further research is certainly considered to be valuable.

As mentioned above, we believed that AEF texture could be used as a biomarker of HCC, so we were trying to find an effective biomarker already in use as the reference. As reported, several biomarkers of HCC have been introduced in literatures, such as AFP, des-gamma carboxy prothrombin, glypican-3, osteopontin, versican, and so on (24–26). However, none of them was optimal (27). In reality, AFP remains the most commonly used biomarker of HCC in the clinic (28–31). The use of AFP has been introduced principally not only for screening, diagnosis, and staging but also for effect prediction, effect monitoring, and prognosis assessment (27, 32, 33). On the other hand, angiogenesis was also reported to be valuable as a biomarker of HCC in clinical trials (34–39). All these existing studies mostly focused on the level of angiogenesis. The level here only meant a mean value, just like AEF was only a mean value of the proportion between arterial feeding and portal feeding. It is kind of an overall description of the tumor rather than an analysis on the tumor's inherent details. Our study showed that AFP was not positively correlated with AEF level but with heterogeneity, which is textural information derived from enhanced images that reflect the inner differences of blood supply changing level in HCC. Our results prove that HCC's angiogenesis differs everywhere inside the tumor. A high variation indicates a lack of regulation and control and suggests a high bioactivity of HCC, which is exactly what we learn AFP can do (27–33). Therefore, we believed that AEF heterogeneity, whether used in combination with AFP or not, could be used as an imaging biomarker of HCC.

After the correlation was confirmed, we performed the regression analysis in order to find the causal relationship between AFP and AEF heterogeneity. Our results showed that they had a two-way causal relationship when AFP was not too high, which meant that HCC angiogenesis and AFP secretion would be probably impacted by each other. However, when AFP was too high, only a one-way cubic causal relationship was observed, which meant that too-high AFP secretion would have a strong positive impact on HCC angiogenesis. This was a sort of impact already reported in other studies (40). This study regarding AFP-producing gastric carcinoma and AFP–antibody treatment also suggested that AFP itself might up-regulate angiogenesis, and the treatment by AFP–antibody could have anti-angiogenic effects. As for the cubic relationship, it might have resulted from the different dimension of AFP and AEF. The serum AFP value in this study was obtained by a blood test, which represented the property of the whole tumor in three dimensions, while AEF was obtained only from a transverse section of the tumor in limited single dimension, whose heterogeneity would, on the contrary, be enlarged cubically.

There were several limitations of this study. First, the lack of sufficient sample size made the grouping a big inequality. Second, the pathological data were not enough because of unavailability in some cases where biopsy was either unaccepted or unnecessary. Third, survival information was not involved because of the complexity of treatment strategy and combination.

## Conclusion

We found that the AEF textures have strong correlations with AFP, the most important biomarker of HCC, indicating that the AEF textures have the potential to reflect the bioactivity of HCC. This finding may enable AEF textures to act as an optional imaging biomarker or assistance to AFP in monitoring HCC during tumor screening, treatment response assessment, or follow-up. Further research and more specific studies with big sample sizes are worthwhile.

## Data Availability Statement

All datasets generated for this study are included in the article/supplementary material.

## Ethics Statement

The studies involving human participants were reviewed and approved by Ethical Committee of Shengjing Hospital. The patients/participants provided their written informed consent to participate in this study. Written informed consent was obtained from the individual(s) for the publication of any potentially identifiable images or data included in this article.

## Author Contributions

XM participated in the study design, cases enrollment, CT acquiring, image processing, clinical management, manuscript writing, and submission. YG participated in CT acquiring, image processing, and statistical analysis. ZL participated in the study design, study review and monitoring, manuscript writing, and submission. FW, HL, and WS participated in cases enrollment and clinical management. All authors contributed to the article and approved the submitted version.

## Conflict of Interest

YG was employed by GE Healthcare (China). The remaining authors declare that the research was conducted in the absence of any commercial or financial relationship that could be construed as a potential conflict of interest.
